# Hybrid Repair *versus* Conventional Open Repair Approaches for Aortic Arch Disease: a Comprehensive Review

**DOI:** 10.21470/1678-9741-2020-0382

**Published:** 2021

**Authors:** Tiago Santos Ribeiro, Hernani de Paiva Gadelha Júnior, Magaly Arrais dos Santos

**Affiliations:** 1 Department of Integrated Medicine, Onofre Lopes University Hospital, Federal University of Rio Grande do Norte, Natal, Brazil.; 2 Dante Pazzanese Institute of Cardiology, São Paulo, SP, Brazil.

**Keywords:** Cardiovascular Surgical Procedures, Aortic, Thoracic, Postoperative Period, Follow-Up Studies

## Abstract

**Objective:**

To investigate whether hybrid repair has supremacy over conventional open repair in aortic arch diseases.

**Methods:**

A comprehensive search was undertaken in two major databases (PubMed and MEDLINE) to identify all studies comparing the two surgical techniques in five years, up to December 2018, that met the established criteria in this study. The search returned 310 papers, and 305 were selected after removing duplicates. The abstracts of the remaining articles were assessed, resulting in 15 studies that went to full-text analysis. After application of the inclusion and exclusion criteria, 8 papers remained for the final revision.

**Results:**

Eight studies met the criteria, with the inclusion of 1,837 patients. From a short-term perspective, hybrid repair and conventional open repair had similar outcomes in terms of postoperative mortality and acute neurological events. Hybrid repair was associated with less respiratory complications and risk of new intervention, as well as reduced hospital length of stay. Conventional open repair showed better mid- and long-term outcomes.

**Conclusion:**

Hybrid repair should be used in selected patients, with a high risk or very high-risk profile for conventional surgery. Finally, since most of the current data were obtained from limited to large samples, with narrow follow-up and had great heterogeneity, the best approach to the aortic arch is still variable. Therefore, the decision of the approach should be individualized and evaluated by the whole Heart Team, considering the expertise of the surgical team.

**Table:** 

Abbreviations, acronyms & symbols	
**COR**	**= Conventional open repair**
**CPB**	**= Cardiopulmonary bypass**
**CTAD**	**= Complex thoracic aortic diseases**
**HCA**	**= Hypothermic circulatory arrest**
**HR**	**= Hybrid repair**
**ICU**	**= Intensive care unit**
**PTFE**	**= Polytetrafluoroethylene**
**TEVAR**	**= Thoracic endovascular aortic repair**

## INTRODUCTION

Complex thoracic aortic diseases (CTAD) are considered a spectrum of diseases that involve the ascending aorta, the aortic arch and descending portions of the aorta and present themselves as a challenge to conventional surgical cardiovascular therapy^[1]^.

Historically, surgical repair of CTAD started with a complete invasive approach, with access through wide-open thoracotomy, use of a synthetic prosthesis to replace all the diseased portion of aorta and reconstruction of the great vessels, in a technique known as complete open aortic arch repair^[1]^. However, the procedure is related to high morbidity and mortality and numerous complications, mainly due to prolonged cardiopulmonary bypass (CPB) time and/or hypothermic circulatory arrest (HCA), a condition required to reduce brain tissue damage and multiorgan ischemia and that can have a direct impact on postoperative neurological outcomes^[2]^. In the last two decades, with the improvement of much less invasive techniques, endovascular repair of the thoracic aorta appeared as an alternative with more desirable outcomes^[(3]^ and marked decrease in morbidity^[4]^.

More recently, hybrid repair (HR), a combination of the open technique in association with the endovascular approach, emerged as a modern treatment modality for properly selected patients^[5]^. The hybrid procedure its fundamentally based in the supra-aortic vessels debranching from the arch to the ascending aorta, creating proximal and distal landing zones suitable for the endovascular insertion of the prosthetic graft^[6]^.

**Fig. 1:**
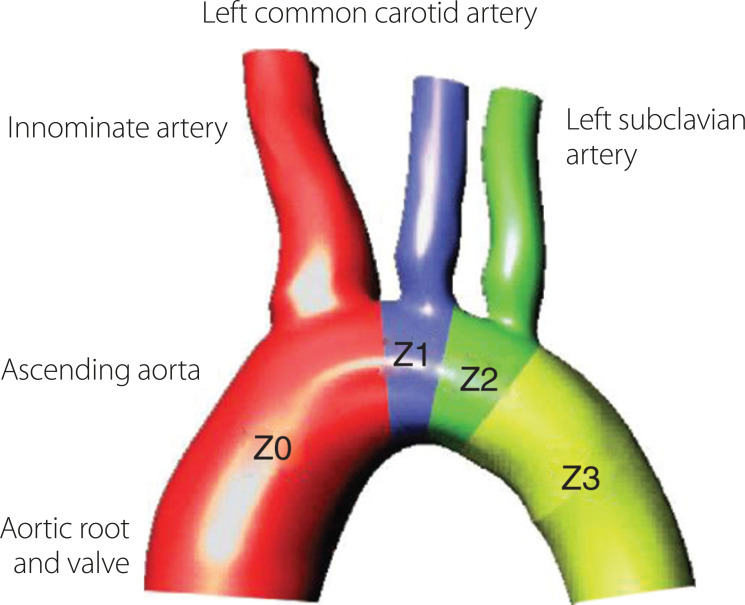
Ishimura classification of zones of the aortic arch: zone (Z) 0, ascending aorta to innominate artery; Z1, innominate artery to left common carotid artery; Z2, left common carotid artery to subclavian artery; Z3, left subclavian artery to proximal descending thoracic aorta. With permission of Rudarakanchana N, Jenkins MP. Hybrid and total endovascular repair of the aortic arch. Br J Surg. 2018;105:315-27^[7]^.

Therefore, anatomical knowledge of the arch is fundamental, which will allow the best approach and the analysis of possible complications. The anatomical variation of the vessels, as well as their angulation, are factors that, in combination with the intense blood flow through the aorta, can make it difficult to release the stent and its duration in a long-term perspective^[7]^. Mitchell et al.^[8]^ described a classification of the aortic arch based in zones, as seen in Figure 1: zone 0, that extends from the proximal ascending aorta to the brachiocephalic trunk; zone 1, that involves the part between the brachiocephalic trunk and the left common carotid artery; zone 2, the segment between the left common carotid artery and the left subclavian artery; zone 3, involving the parts between the left subclavian artery until the proximal descending thoracic aorta; and zone 4, represented by the medium segment of the descending aorta.

**Fig. 2:**
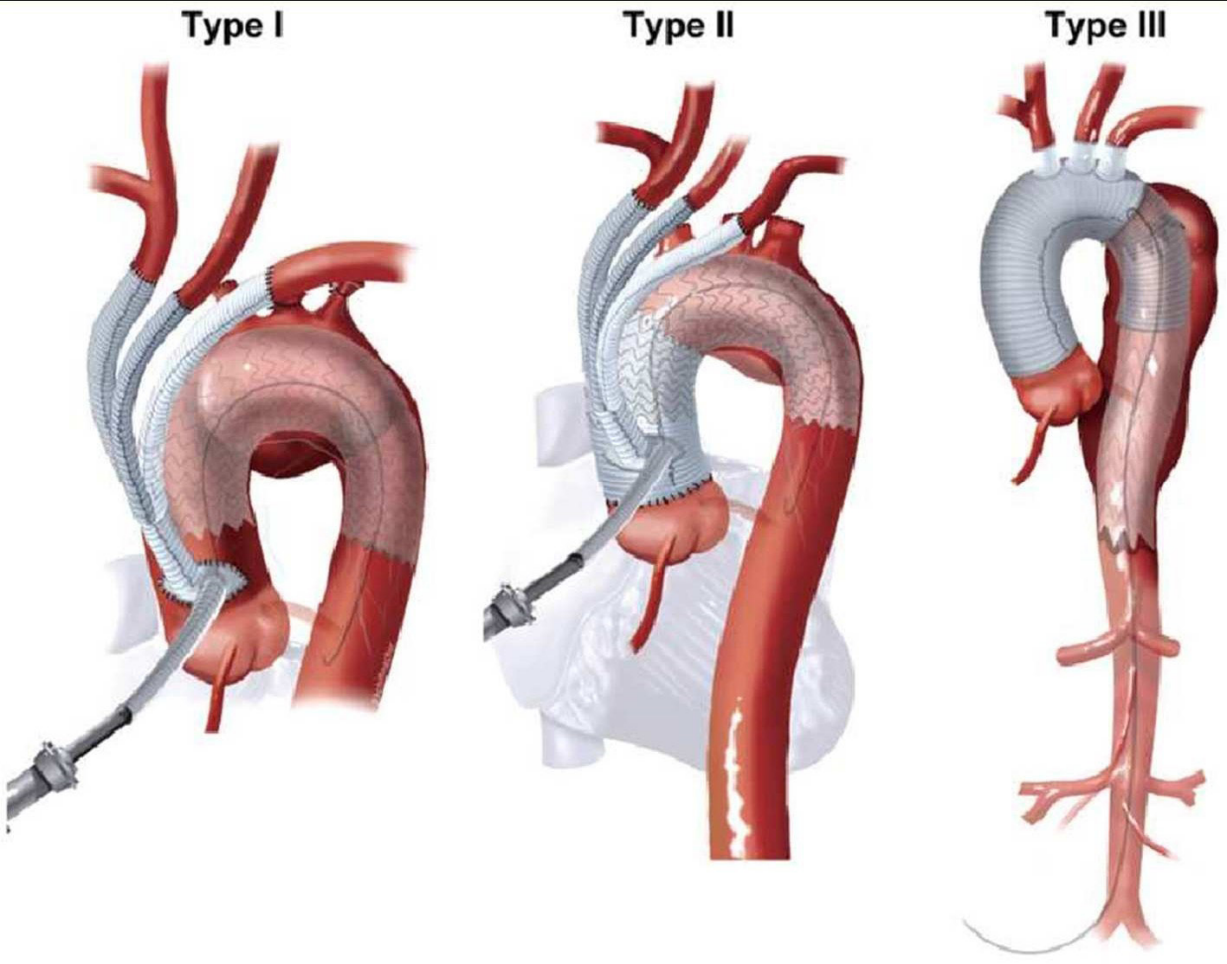
The aortic arch anatomy and the landing zones dictate the type of hybrid arch repair. In a type I hybrid arch, the great vessels are debranched to enable Z0 stent grafting, followed by concurrent antegrade or delayed retrograde TEVAR. For an arch aneurysm without a good proximal Z0, but with an adequate Z3/Z4 distal landing zone, type II hybrid arch repair is performed involving not only the great vessel debranching, but the creation of a proximal Z0 by reconstructing the ascending aorta. More complex aortopathies, such as mega-aorta syndrome, require type III hybrid arch repair. With permission of Vallabhajosyula P, Szeto WY, Desai N, Komlo C, Bavaria JE. Type II arch hybrid debranching procedure. Ann Cardiothorac Surg. 2013;2(3):378-86^[13]^.

There are currently three known types of hybrid repair, in terms of thoracic debranching: types I, II and III, as seen in Figure 2 In type I, the technique involves total arch debranching and creates a landing zone that allows a non-interrupting blood flow to the supra-aortic vessels and, subsequently, endovascular repair. In this technique, an adequate proximal landing zone is required to endoprosthesis delivery^[9]^, in a single-time procedure, with no need of HCA, avoiding, therefore, the risks of postoperative neurological impairment^[10]^. However, the main obstacle of type I hybrid repair is neurological complications^[11]^ with relatively high risks of stroke and endoleaks^[12]^.

In the type II approach, the ascending aorta replacement with a prosthetic graft may be preferred in cases where obtaining an adequate proximal landing zone is not possible due to a diseased aorta, which can be a useful tool to deliver the endovascular stent^[13]^. For a properly tube graft anastomosis, the use of CPB and HCA plus antegrade selective cerebral perfusion is mandatory. Following the procedure, the next step is the supra-aortic vessels debranching, which, however, can be done without aortic clamping. Finally, the endovascular graft is released in an antegrade fashion through the ascending aorta^[14]^.

**Fig. 3:**
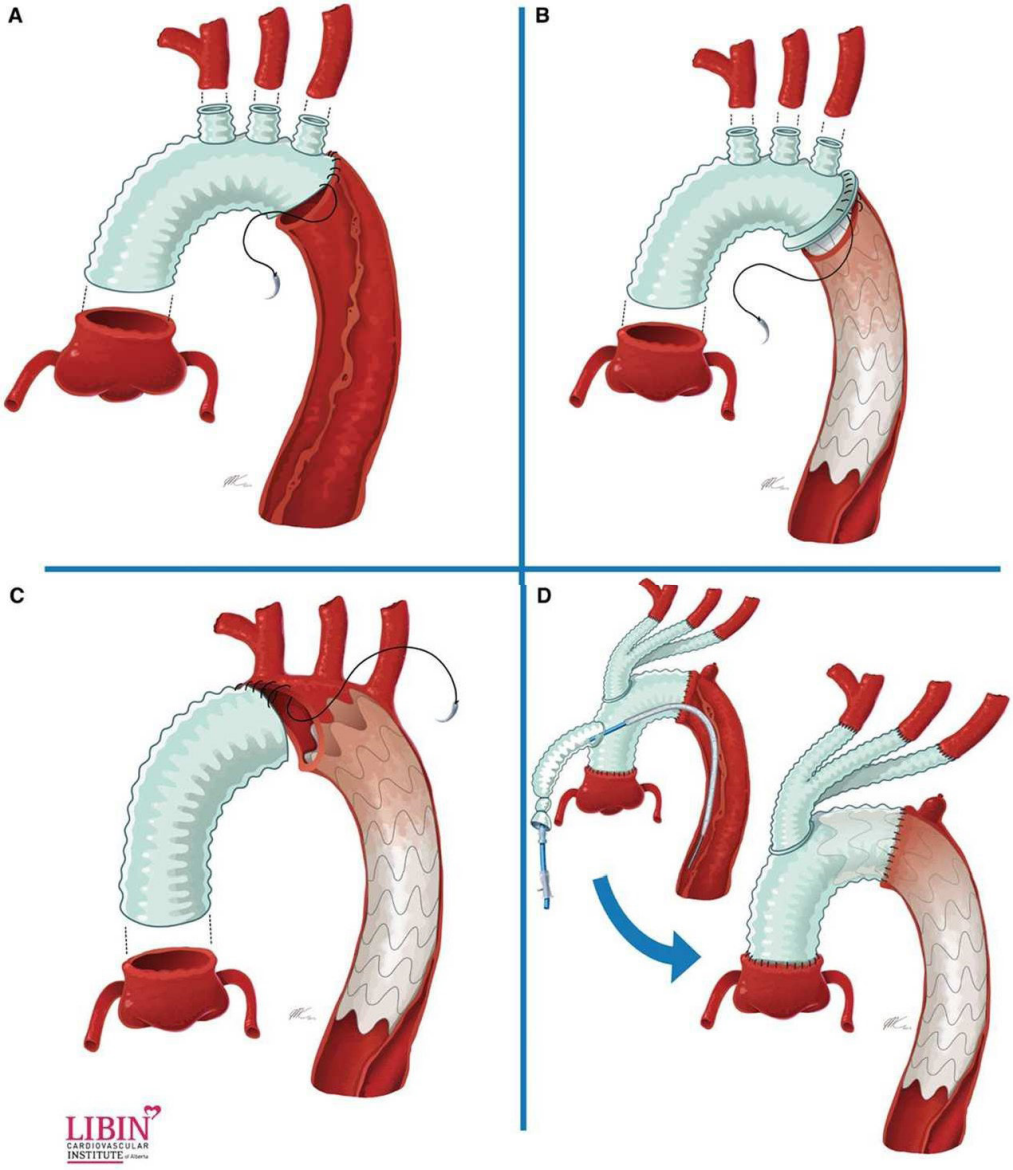
Classification of the extended arch technique. (A) Total aortic arch replacement + standard elephant trunk without descending thoracic aortic stent grafting. (B) Total aortic arch replacement and descending thoracic aortic stent grafting with frozen stent graft placed under circulatory arrest. (C) Hemiarch replacement and descending thoracic aortic stent grafting with the stent graft placed under circulatory arrest. (D) Total aortic arch replacement with stent graft placed after weaning from cardiopulmonary bypass and with the use of fluoroscopy to identify landing zones. With permission of Smith HN, Boodhwani M, Ouzounian M, Saczkowski R, Gregory AJ, Herget EJ, et al. Classification and outcomes of extended arch repair for acute Type A aortic dissection: A systematic review and meta-analysis. Interact Cardiovasc Thorac Surg. 2017;24(3):450-9^[17]^.

The type III repair, known as frozen elephant trunk, is the procedure of choice for patients in whom the aortic disease extends to the ascending aorta, aortic arch, descending and thoracoabdominal portions of aorta, as seen in Figure 3. The proximal aortic replacement is performed through median sternotomy in combination with antegrade insertion of an endograft in the transected aortic arch, in a single-stage procedure, that requires CPB and HCA^[15]^. This single-stage procedure differs from the original conventional open arch repair, described in the 1980s by Dr. Borst^[16]^ in which a double-stage procedure was carried out, but with a high risk of operative mortality, especially in older patients that could not safely stand a second major procedure.

**Fig. 4:**
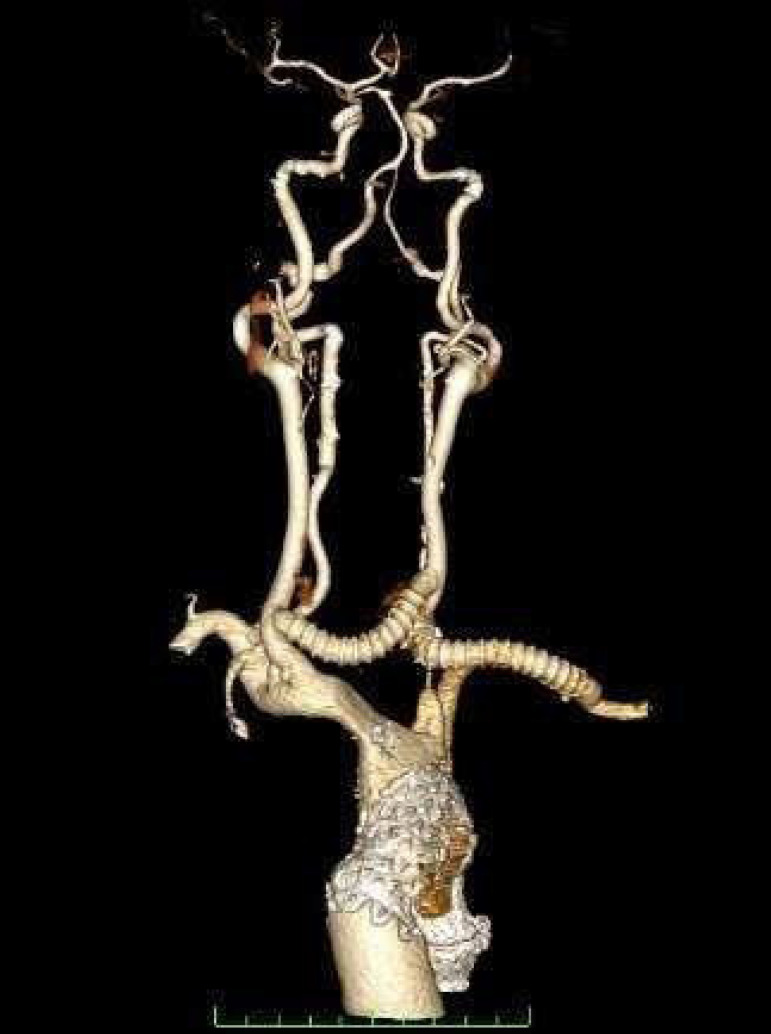
Postoperative CT angiography showing a cervical debranching with reinforced PTFE graft anastomosis from the left subclavian artery to the left carotid artery and from the left carotid artery to the right carotid artery. Image courtesy from the authors.

Furthermore, there are the modalities of cervical debranching that evolved exponentially in the last decade as another option for the treatment of complex aortic arch diseases, especially using fenestrated or branched grafts^[18]^. The aortic arch is anatomically challenging and the use of thoracic endovascular aortic repair (TEVAR) might be difficult in some cases, mainly due to inadequate landing zones for endoprostheses. This situation can be overcome using branched or fenestrated grafts, allowing the debranching to be done remotely. One of the most performed cervical debranching technique involves the transposition of the left subclavian and carotid arteries, as well as more complex cases dealing with the vertebral artery and the right-sided supra-aortic vessels, most commonly the carotid-subclavian artery bypass, as seen in Figure 4^[19]^.

In general, aortic debranching with cervical or supraclavicular incisions is safe and has excellent durability. The main postoperative complications in these cases are neurological. The use of preoperative images, such as angiogram or brain magnetic resonance, to evaluate the vertebral and carotid arterial system, especially in patients with a patent circle of Willis, can guarantee that a unilateral occlusion of one side of the carotid circulation does not cause any cerebral ischemia during the procedure^[19]^.

Branched grafts are built with two interleafed prosthesis, with the “chimney” aspect, and are used to maintain the blood flow of the desired supra-aortic vessels, mainly the brachiocephalic trunk and the left carotid artery. In patients with aneurysms that involve the major portion of the arch or when the stent cannot be properly delivered in the aortic wall, a branched graft is more indicated^[20]^.

Finally, it is possible to conclude that high-risk profile CTAD patients may benefit from hybrid repair, particularly in those with contraindications to the total open repair, with acceptable outcomes in the short and medium term. However, acute neurological events and mortality rates still raise concerns and are considered the “Achilles’ heel” of complex aortic procedures^[13]^.

In view of the above, the authors wanted to recognize what the current literature shows about both interventions, hybrid and open, which is more than necessary, given the rapid development of new technologies. Hence, we aimed to demonstrate, through a comprehensive literature review, the efficacy of HR *versus* total open repair for the treatment of aortic arch diseases.

## METHODS

The study design is a review of articles published in the last 5 years that addressed a comparative analysis between hybrid repair and total open repair for aortic arch disease.

The selected articles were collected from PubMed and MEDLINE databases. The MeSH descriptors chosen were *hybrid repair versus open repair for aortic arch* (with 15 studies found), *hybrid endovascular aortic arch repair* (with 226 studies found) and *total open aortic arch replacement* (with a total of 74 articles).

We included all articles that compared one technique with the other, in adult patients, with samples on any size and followed up in short-, medium- or long-term outcomes, also with the adhesion of retrospective cohorts, in English and in Portuguese. All articles that did not compare the techniques, which included aortic diseases other than the aortic arch, review articles, editorials, randomized clinical trials in progress and meta-analysis were excluded. All abstracts were revised for the application of the inclusion and exclusion criteria. The remaining 15 articles were assessed in the full text, but still did not meet the required criteria. Then, we proceeded to a complete analysis of the eight remaining articles.

**Fig. 5:**
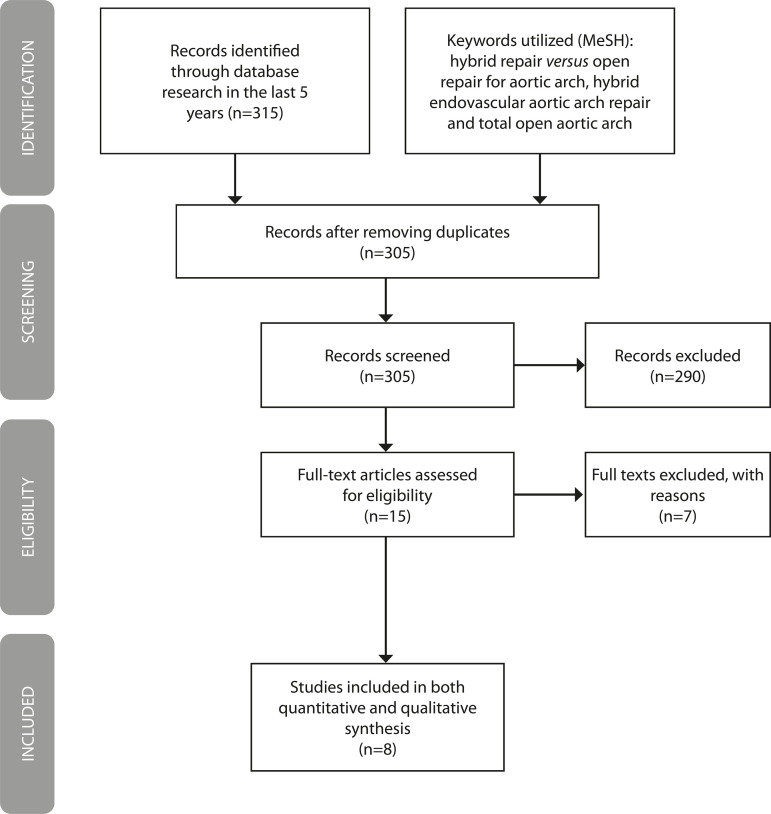
PRISMA flow diagram for review articles.

Then, we proceed to the PRISMA (Preferred Reporting Items for Systematic Reviews and Meta-Analyses) diagram, as shown in Figure 5, for better definition and analysis of the selected articles.

## RESULTS

After proper application of inclusion and exclusion criteria, 8 articles were selected, all in English, as seen in Table 1.

**Table 1:** Summary of conclusions of the eight studies used for the review.

Article/Title	Conclusion
Tokuda et al. Hybrid versus open repair of aortic arch aneurysms: comparison of postoperative and mid-term outcomes with a propensity score-matching analysis	HR with short-term outcomes similar to COR and less CPB and HCA times. In a medium-term perspective, HR is indicated only for high-risk patients.
Preventza et al. Total aortic arch replacement: a comparative study of zone 0 hybrid arch exclusion versus traditional open repair	Both approaches with acceptable outcomes. The individualized approach offers the best option for the patient. Previous heart disease, smoking and congestive heart failure are predictors of worse outcomes.
Joo et al. Comparison of open surgical versus hybrid endovascular repair for descending thoracic aortic aneurysms with distal arch involvement	HR associated with fewer pulmonary complications, but without benefits in terms of mortality and stroke. COR appears to be more trustworthy and long lasting.
Joo et al. Conventional open versus hybrid arch repair of aortic arch disease: early and long-term outcomes	HR with similar results, but COR remains the gold standard therapy for aortic arch diseases. Findings suggest that HR must be reserved for strictly selected patients with significant comorbidities.
Souza et al. Hybrid treatment with complete transposition ofsupra-aortic trunks versus conventional surgery for the treatment of aortic arch aneurysm	Both techniques were similar. However, the sample size was small, which requires further investigation with larger populations.
Iba et al. How should aortic arch aneurysms be treated in the endovascular aortic repair era? A risk-adjusted comparison between open and hybrid arch repair using a propensity score-matching analysis	Surgical outcomes were satisfactory in all groups. Hybrid TEVAR was superior in terms of early surgical recovery, but COR showed more solid results in the long term.
Vikram et al. Open and endovascular repair of the nontraumatic isolated aortic arch aneurysm	The 15-year survival rate was 59%, with late mortality rate predicted due to increasing age, presence of peripheral vascular disease and perioperative stroke. The absence of rupture or reintervention in the aorta was greater after COR.
Yoshitake et al. Comparison of aortic arch repair using the endovascular technique, total arch replacement and staged surgery	The results showed no difference between the procedures, except for early recovery in patients who underwent TEVAR. The long term-survival was similar in all groups, however TEVAR showed inferior reintervention-free rates.

In the study of Tokuda et al.^[3]^, in a sample of 364 individuals, 58 high-risk patients were treated with HR and 124 with COR. Patients treated with HR were older, with more history of malignancy and higher EuroSCORE. Surgical complications were similar in both groups (2.6 *vs*. 0%), with no statistically significant difference. The CPB and HCA times decreased in HR, but without any impact on patient recovery. The final follow-up extended to 52 months and the survival rate free from aortic adverse events were 79% and 99% for HR and COR, respectively.

Preventza et al.^[15]^ performed a multivariate analysis of 16 predictors of adverse outcomes. Of 319 patients, 274 underwent COR and 45 underwent HR with exclusion of the 0-landing zone of the arch. The results showed that operative mortality between the two groups did not differ statistically. Nineteen patients (5.9%) had a permanent stroke, with 15 patients from the conventional repair group (5.5%) and four patients from the HR group (8.9%), with a *P* of 0.32, and two patients, both from the COR group, had permanent paraplegia (*P*=1.00). The HR group had more total neurological complications (*P*=0.051), but not more permanent (*P*=0.32). Previous non-aortic heart disease and congestive heart failure were independent predictors of permanent adverse outcomes, like operative mortality, permanent neurological event or kidney injury. Concomitant coronary artery bypass grafting was predictive of permanent stroke (*P*=0.032), as well as previous cerebrovascular disease. During an average follow-up of 4.5 years, survival rates for the COR group were 78.7%, with no difference between groups (*P*=0.14), even after propensity score matching.

Joo et al.^[21]^ analyzed a cohort of 125 patients diagnosed with descending thoracic aortic aneurysm with distal aortic arch involvement, in which 79 underwent COR and 46 underwent HR in landing zones 1 and 2. Both groups were submitted to propensity score matching, which showed no statistically significant in-hospital mortality (*P*=0.49). The main adverse outcomes were stroke (11,4% *vs*. 8.7%), paraplegia (2,5% *vs*. 0%) and pulmonary complications (19% *vs*. 6.5%), for COR and HR groups, respectively. After adjusting the propensity match, the in-hospital mortality for COR was higher and there was a substantial risk of pulmonary complications. However, both techniques were similar in terms of 30-day mortality, paraplegia and medium-term survival. The 10-year reintervention-free rates were significantly better for COR (85.2%±7.1%) than HR (46.3%±11%; OR=0,13; *P*<0.01).

In another trial, Joo et al.^[22]^ evaluated a sample of 238 patients, of which 174 underwent COR and 64 underwent HR. A retrospective analysis of the clinical outcomes was performed, in addition to the use of a multivariate analysis after propensity score matching. In-hospital mortality rates were 4,6% (8/174) and 6,3% (4/64) in COR and HR groups, respectively, with *P*=0.739. The COR group had a lower incidence of permanent stroke. Overall survival rates in 5 and 10 years were significantly different (COR:87±5.5% and 81.9±4.8%, respectively; HR:69.5±7.4% and 40.8±11.1%, respectively; *P*=0,003). After propensity adjustment, in-hospital mortality and patient comorbidities did not differ significantly. For those in the HR group, a tendency towards permanent stroke was observed (14.5% *vs*. 2.1%, *P*=0.070). In the COR group, the 10-year survival (74.7% *vs*. 42.6%; *P*=0.043) and reintervention-free rates were significantly better (93.2% *vs*. 34%; *P*<0.001).

Souza et al.^[23]^ conducted a study with a small sample of 25 patients, 13 assigned to HR without CPB and 12 patients assigned to COR. Mortality rates were 23% for the HR group and 17% to COR. Postoperative complication rates were similar in both groups.

Iba et al.^[24]^ included 143 and 50 patients admitted for COR and hybrid TEVAR, respectively, for non-dissected aortic arch aneurysms, from 2008 to 2013. The mean values of EuroSCORE II were 4.35±3.65% and 7.78±5.49% for open repair and hybrid TEVAR, respectively. There was no significant early mortality between both groups (4.35±3.65% and 7,78±5,49%). Early morbidity was also equivalent in both groups, but the ICU length of stay was statistically shorter in the hybrid group 4.7 *vs*. 1,6 days, *P*=0.018). During the follow-up, survival rates did not differ statistically (87 *vs*. 81% in 3 years, *P*=0.13), but arch reintervention was required in a patient from the COR group who developed a pseudoaneurysm and in 5 patients from the HR group (4 endoleaks and 1 brachiocephalic artery stenosis). The 3-year reintervention-free rates were 99% in the COR group and 80% in the HR group (*P*<0.001). The propensity score matching showed shorter ICU and hospital length of stay, as well as a greater need for reintervention in the HR group.

Sood et al.^[25]^ had a cohort sample of 2,153 patients who underwent aortic arch repair and, of these, 137 patients, with a median age of 60 years, who were treated with isolated aortic arch resection for non-traumatic aneurysms. COR was assigned to 93 patients, HR to 11 patients and TEVAR for 33 patients, with the last two approaches reserved for high-risk profile candidates who were unable to undergo COR. The propensity score matching and the multivariate analysis were the strategies used to balance the difference in the group of patients, mainly to avoid treatment selection bias. Early mortality rate was observed in 9 patients, defined as in-hospital death or 30-day mortality, and was not statistically different between groups (conventional *vs*. endovascular). Morbidity included stroke, paraplegia, need for dialysis or tracheostomy. The 15-year survival rate was 59%, with late mortality rate predicted due to increasing age, presence of peripheral vascular disease and perioperative stroke (all variables with *P*<0.05). The absence of rupture or reintervention in the aorta was 75% and greater after COR.

Finally, Yoshitake et al.^[26]^ reported a cohort composed of 436 consecutive patients submitted to aortic arch repair, 276 of which were assigned to COR and 118 to hybrid TEVAR. The remaining 42 patients underwent an approach called staged thoracic endovascular aortic repair (STEVAR). The surgical outcomes showed shorter ICU and in-hospital length of stay in the TEVAR group. At 30 days, there was no statistically difference in the groups in terms of neurological outcomes and mortality. In 5 years, survival rates also did not differ statistically.

Table 1 summarizes the main conclusions of the articles in this review.

## DISCUSSION

This review allowed us to select articles of enormous heterogeneity, with large and small samples, and with very diversified outcomes.

Surgical indication for aortic arch diseases might be challenging, since the complexity of the procedure *per se* and the preoperative patient conditioning directly contributes to the postoperative outcomes. Despite the number of studies comparing both techniques, there is still no consensus on the superiority of one approach over the other.

In general, the articles showed better short-term outcomes for hybrid repair, equivalent to conventional open repair, and with a lower incidence of complications, especially due to prolonged CPB use and HCA, as well as pulmonary involvement. Despite this, long-term outcomes suggest better efficacy and durability in patients treated with COR.

Preventza et al.^[15]^ provided clinical data that showed unfavorable outcomes for patients who underwent total arch replacement and had previous diagnose of congestive heart failure, history of smoking and/or previous heart disease. In more recent publications, both techniques had similar results and outcomes in the short term, but in the long term-considered a post-procedure time greater than 5 years-total open aortic repair still remains the gold standard treatment for arch diseases, as noted by Joo et al.^[27]^. In the HR group, a slightly increase in the incidence of permanent neurological deficits was noted, suggesting that cerebrovascular embolic events are a concern even in less invasive approaches.

Another clinical outcome evaluated by one of the studies was related to postoperative pulmonary function complications, which can occur in up to 30% of the patients undergoing thoracotomy to aortic arch repair. Joo et al.^[27]^ showed that respiratory complications were less important in the HR group, which is justified by less manipulation of the lung parenchyma through thoracotomy, in addition to a lower risk of bleeding and edema. Moreover, the prolonged period of mechanical ventilation and tracheostomy had greater impacts on patients in the COR group. Furthermore, the same study did not show benefits in terms of postoperative death and risk of stroke when both procedures were compared.

When compared to TEVAR, COR showed inferiority in terms of post-procedure recovery time, but was superior in terms of long-term results^[24]^. Moreover, the aortic arch morphology remains a decision-making factor for either technique, regardless of age or type of comorbidities^[21]^. Despite being a review with a large sample of patients, our work has few limitations. First, it is complex to generate complete individual patient data from the population, since most articles were retrospective studies. In addition, it is difficult to homogenize the samples, not to mention that retrospection might compromise the statistical quality of the article. On top of that, not all authors used the propensity score matching to diminish the difference between groups. A selection bias might occur, since patients with high operative risk tend to undergo the HR approach than COR, resulting in worse or no higher mortality outcomes. Moreover, most articles had sample sizes in which patients were referred to COR than HR, which may affect the biased outcomes of the hybrid technique.

Finally, another aspect that deserves a spotlight is that the hybrid procedure, due to its relatively new use and indications, is performed with expertise by only few surgeons, which can be another factor that justify the non-superiority of this approach in most of the articles described in this review.

## CONCLUSION

Surgical hybrid repair remains a treatment option for aortic arch disease, with outcomes similar to COR in the short and medium terms of mortality and stroke. It is particularly useful when correctly indicated in a right and individual fashion for each patient, especially for those who presents with high or prohibitive risk for COR. Hybrid procedures with cervical or supraclavicular debranching are also a strategy in patients who cannot undergo thoracic incisions, with safely results reported.

In a long-term perspective, conventional open repair showed more satisfactory results with regard to reintervention, survival rates and morbidity and mortality. That said, COR remains the gold standard treatment for aortic arch disease in patients who can tolerate the open procedure. Finally, the decision-making about one procedure in relation to another must be very well discussed with the Heart Team and the patient. In addition, the center’s experience on performing those techniques should also be considered to provide the best care and minimize poor surgical outcomes and physical and psychological damage.

**Table:** 

Authors' roles & responsibilities	
TSR	Substantial contributions to the conception or design of the work; or the acquisition, analysis, or interpretation of data for the work; agreement to be accountable for all aspects of the work in ensuring that questions related to the accuracy or integrity of any part of the work are appropriately investigated and resolved; final approval of the version to be published
HPGJ	Drafting the work or revising it critically for important intellectual content; agreement to be accountable for all aspects of the work in ensuring that questions related to the accuracy or integrity of any part of the work are appropriately investigated and resolved; final approval of the version to be published
MAS	Drafting the work or revising it critically for important intellectual content; agreement to be accountable for all aspects of the work in ensuring that questions related to the accuracy or integrity of any part of the work are appropriately investigated and resolved; final approval of the version to be published
